# Trends in baseline triglyceride-glucose index and association with predicted 10-year cardiovascular disease risk among type 2 diabetes patients in Thailand

**DOI:** 10.1038/s41598-023-40299-y

**Published:** 2023-08-10

**Authors:** Sethapong Lertsakulbunlue, Mathirut Mungthin, Ram Rangsin, Anupong Kantiwong, Boonsub Sakboonyarat

**Affiliations:** 1 Department of Pharmacology, Phramongkutklao College of Medicine, Bangkok, 10400 Thailand; 2 Department of Parasitology, Phramongkutklao College of Medicine, Bangkok, 10400 Thailand; 3 Department of Military and Community Medicine, Phramongkutklao College of Medicine, Bangkok, 10400 Thailand

**Keywords:** Epidemiology, Endocrine system and metabolic diseases, Public health

## Abstract

Triglyceride-glucose (TyG) index is an independent risk factor for cardiovascular diseases (CVD). Our study determined the trends of the TyG index and its relationship to predicted CVD risk among patients with type 2 diabetes (T2D). A serial cross-sectional study was conducted including 63,815 participants with T2D aged 30–74 years without a history of CVD. The predicted CVD risk was based on the Framingham Heart Study (FHS). The receiver operating characteristic (ROC) curve was utilized for identifying the cutoff point of TyG index to predict intermediate-to-high CVD risk. The relationship between TyG index and predicted CVD risk was tested using linear and logistic regression. Decreasing trends of TyG index were observed between 2014 and 2018 (*p* < 0.001). ROC curve analysis of the TyG index indicated an AUC of 0.57 (95% CI 0.56–0.57, *p* < 0.001) in predicting intermediate-to-high predicted CVD risk, with a cutoff value of TyG index > 9.2 (sensitivity of 55.7%, specificity of 46.8%). An independent relationship between the TyG index and predicted CVD risk was observed. High TyG index was independently associated with intermediate-to-high predicted CVD risk. From our study, the TyG index was positively related to predicted 10-year CVD risk. However, the predictive ability of the TyG index in predicting the intermediate-to-high predicted 10-year CVD risk among patients with T2D remained questionable.

## Introduction

For three decades, deaths from cardiovascular diseases (CVD) have increased proportionally among both men and women^1^. Approximately 17.9 million lives were lost to CVD annually worldwide, and more than one-half of these deaths occurred in Asia^2,3^. Despite the development of advanced prevention, diagnosis, and treatment measures, patients with CVD remain at increased recurrent risk for CVD events. Hence, early CVD risk identification is necessary^4^. Presently, CVD risk assessment tools have been developed to reflect the effects of different interventions and use in different large, randomized, controlled trials^5^. An example of a well-known CVD risk assessment tool is the instrument derived from the Framingham Heart Study (FHS) used to predict 10-year CVD risk^6^.

Insulin resistance (IR) was found to strongly correlate with an increased risk of developing CVD^7^. However, no specific method has been established to determine IR. Currently, the gold standard tests are the euglycemic insulin clamp and intravenous glucose tolerance test, for which both are invasive and expensive; consequently, they are rarely used in clinical practice^8,9^. Nevertheless, in 2008, Simental-Mendia et al. proposed the triglyceride-glucose (TyG) index as a surrogate of IR, and Guerrero-Romero et al. confirmed the hypothesis in 2010^10,11^. The TyG index was also shown superior to the homeostasis model assessment estimated insulin resistance (HOMA-IR) index in assessing IR among patients with and without diabetes^12,13^.

Several cohort studies in the US, China, and Europe have demonstrated the TyG index as an independent risk factor for the incidence of CVD events^14–17^. Moreover, the TyG index has been utilized for identifying the risk of atherosclerosis and was found to be associated with increased coronary artery stenosis. Furthermore, coronary artery calcification, which is a sensitive marker for detecting early atherosclerosis, was independently associated with the presence of a high TyG index^18^.

Recently, the TyG index also shows potential as a marker in predicting CVD risk. A few studies have demonstrated an independent correlation between the TyG index and predicted CVD risk using the FHS equation. In addition, these studies also demonstrated good predictive ability using the TyG index to predict high CVD risk^19,20^. However, several studies failed to support the association between TyG and CVD events among subjects with T2D at baseline^21^. Since the TyG index is a formula composed of fasting TG and FG, it might be affected by hyperlipidemia, diabetes, and related medications. To our knowledge, studies related to the ability of the TyG index to predict CVD risk among patients with diabetes have not been conducted yet.

The present study aimed to explore the relationship between the TyG index and predicted 10-year CVD risk using the FHS equation and identify its cutoff point among patients with T2D. Furthermore, we determined trends of the TyG index among the participants using a database from a Thai DM/HT study^22^. Understanding the relationship might provide further evidence of TyG index application among patients with diabetes.

## Methods

### Study design and subjects

In the present study, we used data retrieved from the database: assessment in Quality of Care among Patients Receiving a Diagnosis with Type 2 Diabetes and Hypertension Visiting the Ministry of Public Health (MoPH) and Bangkok Metropolitan Administration Hospital in Thailand (Thailand DM/HT)^22^. A serial cross-sectional study was conducted in 2014, 2015, and 2018, after receiving permission from the National Health Security Office (NHSO) and the Medical Research Network of the Consortium of Thai Medial Schools (MedResNet). The study design and data collection protocols of Thailand DM/HT have been published^22^. The study constitutes a cross-sectional study among Thai adult patients with T2D or HT including all MoPH Hospitals, Bangkok Metropolitan Hospitals, and public and private clinics under the NHSO program nationwide. A total of 33,288, 32,616, and 36,793 patients with T2D were recruited in 2014, 2015, and 2018, respectively^22^.

**Figure 1:**
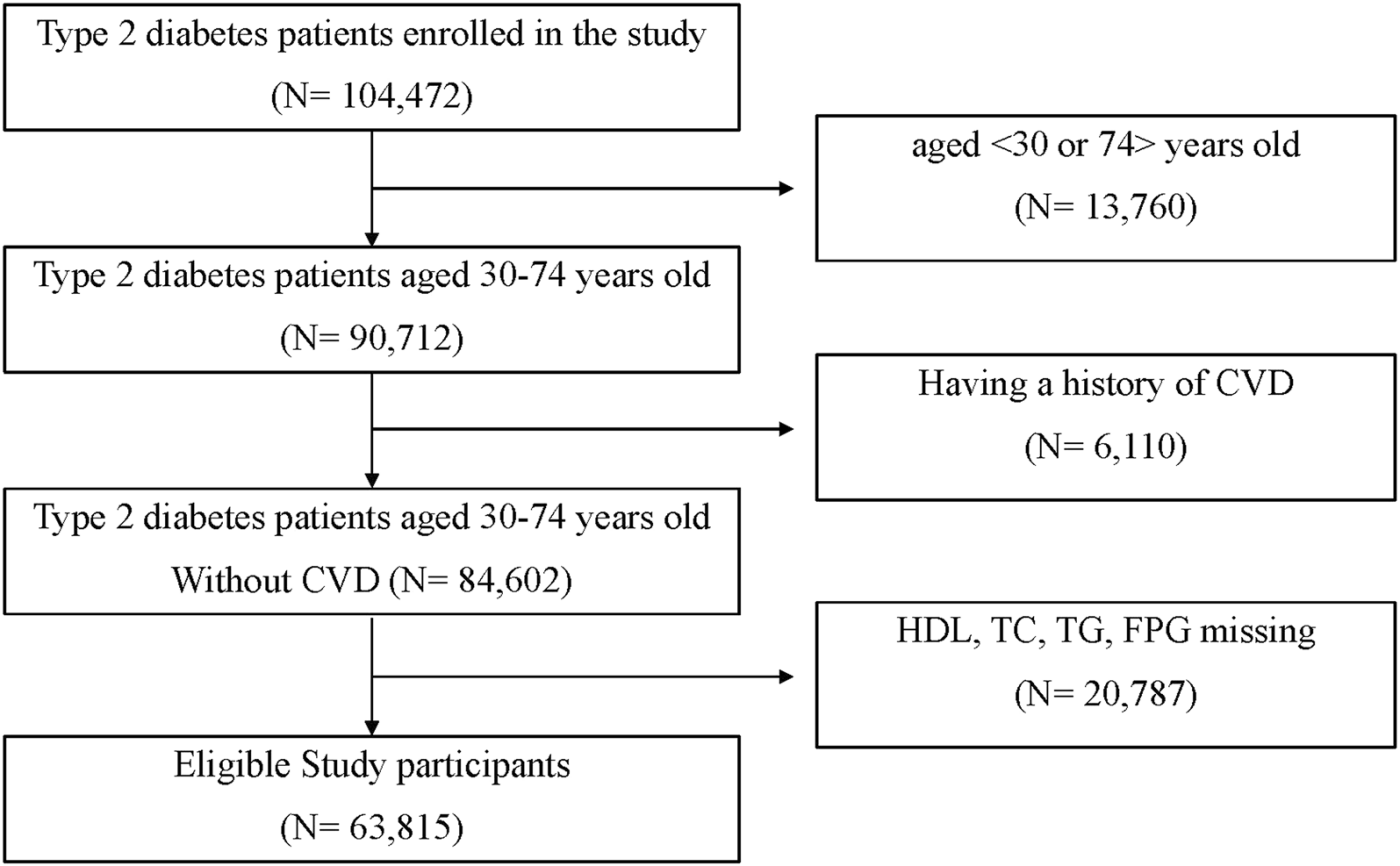
Flow of enrolled patients with type 2 diabetes receiving care in Thailand in 2014, 2015, and 2018.

Regarding the predicted 10-year CVD risk calculation based on the FHS algorithm^6^, we included patients with T2D aged 30–74 years in the present study. In addition, patients with T2D having a history of CVD (coronary death, myocardial infarction, coronary insufficiency, angina, ischemic stroke, hemorrhagic stroke, transient ischemic attack, peripheral artery disease, and heart failure) were excluded. Participants with absent laboratory exams included in the FHS algorithm and TyG index were also excluded (HDL, TC, TG, and FPG). Thus, our final analytic sample included 63,815 participants (Fig. 1).

### Data collection

A standardized case report form (CRF) was utilized for medical records, completed by health care personnel (usually a well-trained registered nurse) using a standard protocol, and then sent to the MedResNet central data management unit in Nonthaburi, Thailand. Data, collected from the patient's medical records, included baseline information, current medications, laboratory testing results, and the status of diabetes complications. The predicted 10-year risk for CVD was calculated using the risk algorithm derived from the FHS data among individuals without a history of CVD (coronary heart disease, stroke, peripheral artery disease, or heart failure)^6^. According to the laboratory-based data in predicting 10-year CVD risk, the variables in the algorithm included age, sex, systolic blood pressure (SBP), history of current smoking, history of diabetes, treatment for HT, total cholesterol (TC), and high-density lipoprotein (HDL) cholesterol. The intermediate predicted 10-year CVD risk was defined as a predicted 10-year CVD risk of 10 to 20%, and > 20% indicated a high predicted 10-year CVD risk^6^. The equation, ln [TG (mg/dL) *FBG (mg/dL)/2], was utilized for calculating the TyG index^4^.

### Statistical analysis

All analyses were performed using StataCorp, 2021, *Stata Statistical Software: Release 17*. College Station, TX: StataCorp LLC. Frequency distribution of demographic characteristics was carried out to describe the study subjects. Categorical data were presented as percentages. Continuous variables were presented as mean and standard deviation (SD) or median and interquartile range (IQR) as appropriate. For trend, age- and sex-adjusted means of TyG index were calculated using data for 2014, 2015, and 2018. Linear regression was employed for age- and sex-adjusted means to test the statistical significance of linear and nonlinear trends. The nonlinear trend was first tested by adding a quadratic term in the regression model. When the result was insignificant, a linear trend was tested. Log-transformation was performed to the predicted 10-year CVD risk (nonnormal distribution, linear assumption was violated). Normal Q-Q plot and histogram were used to check for normality. The Breusch–Pagan test was utilized for assessing homoscedasticity. Linear regression analysis was then used to determine the linear association between the TyG index and log-transformed predicted 10-year CVD risk (laboratory-based). Youden’s J statistics of the ROC curve were utilized for identifying the TyG cutoff point in predicting intermediate-to-high CVD risk, and the area under the ROC curve (AUC) was calculated. Binary logistic regression analysis was conducted to determine the odds ratio (OR) with 95% CI. Potential confounders were analyzed using univariable logistic regression analysis (shown in Supplementary Table S1). Furthermore, any variables with multicollinearity or already included in the variable of interest (TyG index) and outcome (predicted 10-year CVD risk), except for age and sex, were excluded from the multivariable analysis. Multivariable analysis was performed using logistic regression analysis, and adjusted odds ratio (AOR) was presented. All statistical tests were two-sided, and a *p*-value less than 0.05 was considered statistically significant.

### Ethics approval and consent to participate

The study was approved by the Medical Department Ethic Review Committee for Research in Human Subjects, Institutional Review Board, RTA (approval no. S072h/65_Exp), in accordance with the international guidelines including the Declaration of Helsinki, the Belmont Report, CIOMS Guidelines, and the International Conference on Harmonization of Technical Requirements for Registration of Pharmaceuticals for Human Use–Good Clinical Practice (ICH-GCP). Due to using secondary data, a waiver of documentation of informed consent was utilized, and the waiver for informed consent was granted by the Institutional Review Board, RTA Medical Department.

## Results

### Baseline characteristics of participants

A total of 63,815 participants with T2D aged 30–74 years without history of CVD were enrolled. Table 1 presents the baseline characteristics of the participants according to the year of enrollment. The average age was 58.9 ± 9 years, and about one-third of the participants were males. Among all, 33.2, 30.8, 16.2, 15.7, and 4.1% of participants resided in the central, northeast, south, north regions, and Bangkok, respectively. About three-quarters of participants were under the universal health coverage scheme and received T2D care at community hospitals. Overall, the participants' fasting plasma glucose median (IQR) was 143 (120–176) mg/dL. The median (IQR) fasting triglyceride levels in participants decreased from 149 (107–210) mg/dL in 2014 to 142 (101–200) mg/dL in 2018. However, a significant increase was observed in the 10-year predicted laboratory-based CVD risk median (IQR) from 18.5 (11.5–28.9)% in 2014 to 19.6 (12.6–30.0)% in 2018. Nevertheless, the TyG index mean remained constant at 9.3 ± 0.6 each year.

**Table 1:** Demographic characteristics of participants (N = 63,815).

Year	2014	2015	2018
Characteristics	n = 19,070	n = 19,434	n = 25,311
	n (%)	n (%)	n (%)
Sex			
Male	5669 (29.7)	6173 (31.8)	8269 (32.7)
Female	13,401 (70.3)	13,261 (68.2)	17,042 (67.3)
Age (years)			
30–39	569 (3.0)	514 (2.6)	565 (2.2)
40–49	2847 (14.9)	2617 (13.5)	3056 (12.1)
50–59	6444 (33.8)	6570 (33.8)	8315 (32.9)
60–69	6998 (36.7)	7296 (37.5)	10,066 (39.8)
70–74	2212 (11.6)	2437 (12.5)	3309 (13.1)
Mean ± SD	58.4 ± 9.1	58.8 ± 9.1	59.3 ± 8.9
Geographic region			
Northeast	6941 (36.4)	5963 (30.7)	6757 (26.7)
North	2762 (14.5)	3021 (15.5)	4226 (16.7)
Central	5816 (30.5)	6661 (34.3)	8696 (34.4)
South	2896 (15.2)	3020 (15.5)	4415 (17.4)
Bangkok	655 (3.4)	769 (4.0)	1217 (4.8)
Hospital level			
Regional hospital (S/A)	1454 (7.6)	1845 (9.5)	1737 (6.9)
General hospital	2785 (14.6)	3189 (16.4)	3748 (14.8)
Community hospital	14,831 (77.8)	14,400 (74.1)	19,826 (78.3)
Occupation			
Outdoor	11,728 (63.9)	11,619 (61.8)	14,552 (59.6)
Indoor	2222 (12.1)	2663 (14.2)	3320 (13.6)
Unemployed	4404 (24.0)	4533 (24.1)	6555 (26.8)
Scheme			
Universal healthcare coverage	15,021 (78.9)	14,952 (76.9)	19,926 (78.7)
Civil servant medical benefit	2859 (15.0)	3223 (16.6)	3891 (15.4)
Social security	897 (4.7)	859 (4.4)	1158 (4.6)
Others	255 (1.3)	400 (2.1)	336 (1.3)
Current smoker	925 (5.1)	871 (4.6)	989 (4.0)
Hypertension	14,114 (74.0)	14,855 (76.4)	19,102 (75.5)
Dyslipidemia	13,429 (70.4)	14,483 (74.5)	18,069 (71.4)
Statin used	11,305 (59.3)	11,903 (61.2)	17,370 (68.6)
Fibrate used	2887 (15.1)	2488 (12.8)	1845 (7.3)
Insulin used	4176 (21.9)	4402 (22.7)	5514 (21.8)
DM duration (years)			
Median (IQR)	6.0 (4.0–9.0)	7.0 (4.0–10.0)	7.0 (4.0–11.0)
BMI (kg/m^2^)			
Mean ± SD	25.8 ± 4.5	26 ± 4.6	26.1 ± 4.7
Last SBP			
Mean ± SD	129.4 ± 15.6	131.1 ± 15.6	132.4 ± 14.7
Last DBP (mean ± SD)			
Mean ± SD	74.6 ± 10.0	75.1 ± 10.0	75.3 ± 9.9
FPG (mg/dL) (median (IQR))			
Median (IQR)	143.0 (120.0–177.0)	143.0 (120.0–176.0)	143.0 (121.0–175.0)
HDL (median (IQR))			
Median (IQR)	45.0 (38.0–54.0)	46.0 (38.0–54.0)	47.0 (39.0–56.0)
Total cholesterol (median (IQR))			
Median (IQR)	184.0 (158.0–214.0)	182.0 (156.0–213.0)	180.0 (154.0–209.0)
Triglyceride (median (IQR))			
Median (IQR)	149.0 (107.0–210.0)	145.0 (104.0–203.0)	142.0 (101.0–200.0)
Predicted 10-year CVD risk (laboratory-based)			
Mean ± SD	22.0 ± 14.4	22.8 ± 14.4	22.9 ± 14.0
Median (IQR)	18.5 (11.5–28.9)	19.5 (12.2–29.8)	19.6 (12.6–30.0)
Triglyceride glucose index			
Mean ± SD	9.3 ± 0.6	9.3 ± 0.6	9.3 ± 0.6
Median (IQR)	9.3 (8.9–9.7)	9.3 (8.9–9.7)	9.2 (8.8–9.7)

*SD* standard deviation, *IQR* interquartile range, *DM* diabetes mellitus, *BMI* body mass index, *SBP* systolic blood pressure, *DBP* diastolic blood pressure, *FPG* fasting plasma glucose, *HDL* high-density lipoprotein, *LDL* low-density lipoprotein, *CVD* cardiovascular diseases.

### Trends in mean TyG index among patients with T2D in Thailand from 2014 to 2018

Table 2 presents the trend of age- and sex-adjusted mean TyG index. The overall age- and sex-adjusted mean TyG index among patients with T2D decreased from 9.32 (95% CI 9.31–9.33) in 2014 to 9.28 (95% CI 9.27–9.29) in 2018 (*p* < 0.001). The age-adjusted mean TyG index decreased among both males and females (*p* < 0.001). The highest sex-adjusted mean TyG index was observed in the age group of 30–39 years, remaining unchanged and ranging from 9.42 to 9.45 (*p* = 0.437) and decreasing as age group increased. The sex-adjusted mean TyG index seemed to gradually decrease in older age groups. Reduced TyG trends were observed in age groups between 40 and 70 years (*p* < 0.05). Stratified by region, the age- and sex-adjusted mean TyG index decreased only in the central region (*p* < 0.001). Meanwhile, the highest age- and sex-adjusted means of the TyG index were observed in the northeast region, ranging between 9.40 and 9.43 in 2014–2018 (*p* for trend = 0.067).

**Table 2:** The trends in the overall age- and sex-adjusted and age-adjusted means of triglyceride-glucose index among Thai patients with type 2 diabetes from 2014 to 2018.

Year	2014			2015			2018			*p* for trends
	n	Means	95% CI	n	Means	95% CI	n	Means	95% CI	
Total^a^	19,070	9.32	9.31–9.33	19,434	9.29	9.28–9.3	25,311	9.28	9.27–9.29	< 0.001^d^
Sex^b^										
Male	5669	9.32	9.31–9.34	6173	9.29	9.27–9.30	8269	9.27	9.26–9.28	< 0.001^d^
Female	13,401	9.32	9.31–9.33	13,261	9.30	9.28–9.31	17,042	9.28	9.27–9.29	< 0.001^d^
Age (years)^c^										
30–39	569	9.45	9.40–9.51	514	9.44	9.38–9.50	565	9.42	9.36–9.48	0.437
40–49	2847	9.41	9.38–9.43	2617	9.38	9.35–9.40	3056	9.36	9.34–9.39	0.005^d^
50–59	6444	9.36	9.35–9.38	6570	9.32	9.30–9.33	8315	9.32	9.30–9.33	< 0.001^d^
60–69	6998	9.29	9.28–9.31	7296	9.26	9.25–9.27	10,066	9.24	9.23–9.25	< 0.001^d^
70–74	2212	9.20	9.17–9.22	2437	9.20	9.17–9.22	3309	9.17	9.15–9.19	0.059
Geographic region^a^										
Northeast	6941	9.43	9.41–9.44	5963	9.40	9.39–9.42	6757	9.40	9.39–9.42	0.067
North	2762	9.29	9.26–9.31	3021	9.23	9.21–9.26	4226	9.24	9.22–9.26	0.062
Central	5816	9.27	9.25–9.28	6661	9.25	9.24–9.27	8696	9.22	9.21–9.23	< 0.001^d^
South	2896	9.26	9.24–9.28	3020	9.23	9.21–9.26	4415	9.25	9.23–9.27	0.920
Bangkok	655	9.20	9.15–9.24	769	9.24	9.20–9.29	1217	9.23	9.20–9.27	0.457

The *p* for trend is calculated by adding a quadratic term in the regression model. When the result was insignificant, a linear trend was tested. The level of statistical significance is set at *p* for trend < 0.05.

*CI* confidence interval.

^a^Age- and sex-adjusted mean using regression analyses.

^b^Age-adjusted mean using regression analyses.

^c^Sex-adjusted mean using regression analyses.

^d^Nonlinear trend.

### Relationship between predicted 10-year risk for CVD based on laboratory data and the TyG index

#### Linear relationship between the TyG index and log-transformed predicted 10-year risk for CVD based on laboratory data

Table 3 shows linear regression analysis demonstrating a positive relationship between the TyG index and log-transformed predicted 10-year risk for CVD. The adjusted β coefficient was 0.21 (95% CI 0.21–0.22, *p* < 0.001). The TyG index was divided into quartiles. After adjusting for age, sex, BMI, region, scheme, hospital level, year enrolled, occupation, HT comorbidity, DKD, insulin used, fibrate used, and statin used, gradual increase in adjusted β coefficient was observed in higher quartiles. For the 4th quartile, the adjusted β coefficient was 0.33 (95% CI 0.32–0.34, *p* < 0.001) in comparison with the 1st quartile. Similarly, after stratified by sex, Table 4 demonstrates an independent linear association between the TyG index and predicted CVD risk [adjusted β coefficient of 0.20 (95% CI 0.19–0.21) and 0.22 (95% CI 0.21–0.22), respectively, among males and females, *p* < 0.001]. Table 4 also presents comparable results despite being stratified by age groups of 30–44, 45–59, and 60–74 years. The highest relationship was shown in the age group of 45–59 years (adjusted β = 0.21; 95% CI 0.20–0.22, *p* < 0.001).

**Table 3:** Linear regression analysis of CVD risk and triglyceride-glucose index among Thai patients with type 2 diabetes from 2014 to 2018.

Variables	Univariate			Multivariate*		
	β Coefficient	95% CI	*p*-value	Adjusted β	95% CI	*p*-value
Log-transformed predicted 10-year CVD risk						
TyG index	0.155	0.147–0.163	< 0.001	0.211	0.205–0.216	< 0.001
TyG index (Quartiles)						
Quartile 1	Ref			Ref		
Quartile 2	0.097	0.083–0.112	< 0.001	0.119	0.110–0.128	< 0.001
Quartile 3	0.158	0.143–0.173	< 0.001	0.209	0.200–0.218	< 0.001
Quartile 4	0.240	0.226–0.255	< 0.001	0.329	0.320–0.338	< 0.001

*TyG* triglyceride-glucose, *CVD* cardiovascular diseases, *CI* confidence interval.

*Adjusted for age, sex, body mass index, region, scheme, hospital level, year, occupation, hypertension comorbidity, diabetic kidney disease, insulin used, fibrate used, and statin used.

**Table 4:** Sex-specific and age-specific multivariable linear regression analysis of CVD risk and triglyceride-glucose index among Thai patients with type 2 diabetes from 2014 to 2018.

Variables	Multivariate		
	Adjusted β	95% CI	*p*-value
Log-transformed predicted 10-year CVD risk			
Male*			
TyG index	0.203	0.194–0.211	< 0.001
TyG index (quartiles)			
Quartile 1 (< 8.86)	Ref		
Quartile 2 (8.86–9.26)	0.122	0.108–0.137	< 0.001
Quartile 3 (9.26–9.69)	0.218	0.203–0.232	< 0.001
Quartile 4 (> 9.69)	0.323	0.308–0.339	< 0.001
Female*			
TyG index	0.215	0.208–0.221	< 0.001
TyG index (quartiles)			
Quartile 1 (< 8.86)	Ref		
Quartile 2 (8.86–9.26)	0.118	0.107–0.129	< 0.001
Quartile 3 (9.26–9.69)	0.205	0.194–0.216	< 0.001
Quartile 4 (> 9.69)	0.332	0.321–0.343	< 0.001
Age 30–44**			
TyG index	0.193	0.171–0.216	< 0.001
TyG index (quartiles)			
Quartile 1 (< 8.86)	Ref		
Quartile 2 (8.86–9.26)	0.099	0.054–0.145	< 0.001
Quartile 3 (9.26–9.69)	0.228	0.185–0.271	< 0.001
Quartile 4 (> 9.69)	0.324	0.281–0.367	< 0.001
Age 45–59**			
TyG index	0.208	0.199–0.217	< 0.001
TyG index (quartiles)			
Quartile 1 (< 8.86)	Ref		
Quartile 2 (8.86–9.26)	0.119	0.103–0.135	< 0.001
Quartile 3 (9.26–9.69)	0.207	0.191–0.223	< 0.001
Quartile 4 (> 9.69)	0.322	0.306–0.338	< 0.001
Age 60–74**			
TyG index	0.193	0.186–0.201	< 0.001
TyG index (quartiles)			
Quartile 1 (< 8.86)	Ref		
Quartile 2 (8.86–9.26)	0.115	0.103–0.127	< 0.001
Quartile 3 (9.26–9.69)	0.195	0.183–0.208	< 0.001
Quartile 4 (> 9.69)	0.297	0.284–0.310	< 0.001

*TyG* triglyceride-glucose, *CVD* cardiovascular diseases, *CI* confidence interval.

*Adjusted for age, body mass index, region, scheme, hospital level, year, occupation, hypertension comorbidity, diabetic kidney disease, insulin use, fibrate use, and statin use.

**Adjusted for sex, body mass index, region, scheme, hospital level, year, occupation, hypertension comorbidity, diabetic kidney disease, insulin use, fibrate use, and statin use.

#### Receiver operating characteristics curve analysis of the TyG index prediction of intermediate-to-high predicted 10-year risk for CVD

**Figure 2:**
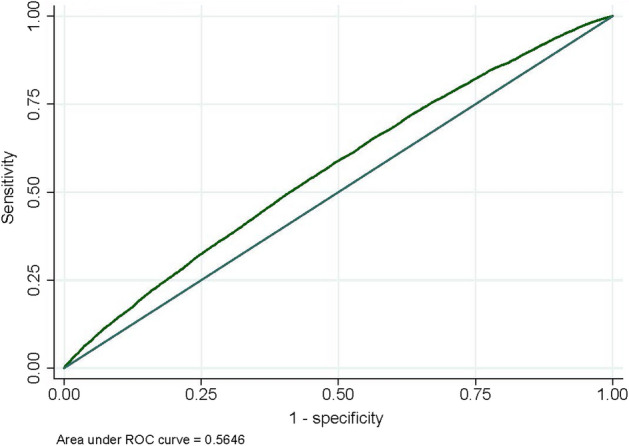
ROC curve of the TyG index to predict intermediate-to-high 10-year predicted CVD risk.

Figure 2 illustrates the ability of the TyG index to predict intermediate-to-high predicted 10-year risk for CVD among patients with T2D. The area under the AUC predicted by TyG was 0.57 (95% CI 0.56–0.57, *p* < 0.001). The results of the ROC analysis curve demonstrated that a TyG of 9.20 (sensitivity 56%, specificity 47%) exhibited the highest Youden’s index for determining intermediate-to-high predicted 10-year risk for CVD among patients with T2D and proved the most effective cutoff value in our study. The ROC analysis curve differentiated by sex is also shown in Supplementary Fig. S1, demonstrating an AUC of 0.57 (95% CI 0.55–0.58, *p* < 0.001) among males and 0.57 (95% CI 0.56–0.58,* p* < 0.001) among females.

#### Logistic regression analysis between the TyG index and intermediate-to-high predicted 10-year risk for CVD based on laboratory data

After adjusting for potential confounders, a single unit increase of the TyG index escalated the odds of intermediate-to-high predicted 10-year CVD risk by 2.54 times (95% CI 2.41–2.68, *p* < 0.001). A TyG index of 9.20 and above also increased the risk of having intermediate-to-high predicted CVD risk (AOR; 2.50, 95% CI 2.35–2.66, *p* < 0.001) as shown in Table 5. After stratified by sex, a higher risk of intermediate-to-high predicted 10-year CVD risk was observed among males with a high TyG index in comparison with females with a high TyG index (Table 6); both were significant (*p* < 0.001). In Table 6, stratification by age was also performed, and a higher TyG index was shown to be an independent determinant of intermediate-to-high CVD risk. Logistic regression analysis between the TyG index and high predicted 10-year risk for CVD, based on laboratory data, was also performed, and similar results are shown in Supplementary Table S2.

**Table 5:** Logistic regression analysis of intermediate-to-high CVD risk and triglyceride-glucose index among Thai patients with type 2 diabetes from 2014 to 2018.

Variables	Total					
	OR	95% CI	*p*-value	*AOR	95% CI	*p*-value
Intermediate to high predicted 10-year CVD risk						
TyG index	1.46	1.42–1.52	< 0.001	2.54	2.41–2.68	< 0.001
TyG index						
TyG index ≤ 9.2	Ref			Ref		
TyG index > 9.2	1.43	1.37–1.49	< 0.001	2.50	2.35–2.66	< 0.001
TyG index (quartiles)						
Quartile 1	Ref			Ref		
Quartile 2	1.32	1.24–1.39	< 0.001	1.71	1.58–1.85	< 0.001
Quartile 3	1.49	1.40–1.57	< 0.001	2.59	2.39–2.81	< 0.001
Quartile 4	1.79	1.68–1.90	< 0.001	4.32	4.00–4.72	< 0.001

*OR* odds ratio, *AOR* adjusted odds ratio, *CI* confidence interval, *TyG* triglyceride-glucose, *CVD* cardiovascular diseases.

*Adjusted for age, sex, body mass index, region, scheme, hospital level, year, occupation, hypertension comorbidity, diabetic kidney disease, insulin used, fibrate used, and statin used.

**Table 6:** Sex-specific and age-specific multivariable logistic regression analysis of intermediate-to-high CVD risk and triglyceride-glucose index among Thai patients with type 2 diabetes from 2014 to 2018.

Variables	Male*			Female*			Age 30–44**			Age 45–59**			Age 60–74**		
	AOR	95% CI	*p*-value	AOR	95% CI	*p*-value	AOR	95% CI	*p*-value	AOR	95% CI	*p*-value	AOR	95% CI	*p*-value
Intermediate to high predicted 10-year CVD risk															
TyG index	2.80	2.43–3.21	< 0.001	2.52	2.38–2.66	< 0.001	2.30	2.01–2.64	< 0.001	2.17	2.05–2.30	< 0.001	2.63	2.34–2.96	< 0.001
TyG index															
TyG index ≤ 9.2	Ref			Ref			Ref			Ref			Ref		
TyG index > 9.2	3.25	2.72–3.87	< 0.001	2.41	2.25–2.57	< 0.001	2.83	2.35–3.42	< 0.001	2.09	1.95–2.24	< 0.001	2.44	2.13–2.80	< 0.001
TyG index (quartiles)															
Quartile 1 (< 8.86)	Ref			Ref			Ref			Ref			Ref		
Quartile 2 (8.86–9.26)	1.55	1.24–1.93	< 0.001	1.73	1.59–1.89	< 0.001	1.37	1.03–1.83	0.033	1.56	1.43–1.71	< 0.001	1.82	1.56–2.13	< 0.001
Quartile 3 (9.26–9.69)	3.01	2.37–3.82	< 0.001	2.53	2.32–2.77	< 0.001	2.60	1.99–3.39	< 0.001	2.16	1.97–2.37	< 0.001	2.70	2.26–3.22	< 0.001
Quartile 4 (> 9.69)	5.41	4.20–6.97	< 0.001	4.19	3.80–4.61	< 0.001	4.30	3.31–5.57	< 0.001	3.24	2.93–3.57	< 0.001	4.19	3.36–5.23	< 0.001
Year															
2014	Ref			Ref			Ref			Ref			Ref		
2015	0.89	0.72–1.11	0.296	1.20	1.10–1.29	< 0.001	0.91	0.74–1.12	0.371	1.21	1.11–1.31	0.003	1.14	0.97–1.34	0.116
2018	0.69	0.56–0.85	< 0.001	1.20	1.12–1.30	< 0.001	0.84	0.69–1.03	0.098	1.18	1.09–1.28	0.001	1.24	1.06–1.44	0.006
Body mass index	1.07	1.05–1.09	< 0.001	1.04	1.03–1.05	< 0.001	1.02	1.00–1.03	0.019	1.02	1.02–1.03	< 0.001	1.02	1.01–1.04	0.003
Geographic region															
Northeast	Ref			Ref			Ref			Ref			Ref		
North	1.06	0.83–1.36	0.644	1.13	1.03–1.24	0.011	0.97	0.74–1.26	0.803	1.07	0.97–1.18	0.186	1.15	0.94–1.40	0.183
Central	1.16	0.93–1.44	0.188	1.06	0.98–1.15	0.177	1.09	0.87–1.36	0.466	1.04	0.96–1.14	0.333	1.21	1.03–1.42	0.023
South	1.44	1.11–1.87	0.006	1.29	1.17–1.42	< 0.001	1.45	1.13–1.85	0.003	1.18	1.07–1.31	0.001	1.20	0.99–1.46	0.067
Bangkok	0.94	0.61–1.45	0.766	1.38	1.10–1.72	0.005	1.39	0.86–2.24	0.179	1.17	0.92–1.48	0.197	1.27	0.83–1.93	0.266
Hospital level															
Regional hospital (S/A)	Ref						Ref			Ref			Ref		
General hospital	1.80	1.28–2.53	0.001	1.24	1.07–1.43	0.004	1.56	1.07–2.26	0.019	1.27	1.08–1.48	0.003	1.13	0.86–1.49	0.364
Community hospital	1.54	1.16–2.05	0.003	1.33	1.17–1.51	< 0.001	1.28	0.92–1.78	0.136	1.26	1.10–1.45	0.001	1.38	1.09–1.76	0.009
Occupation															
Outdoor	Ref			Ref			Ref			Ref			Ref		
Indoor	0.97	0.78–1.21	0.799	1.12	1.00–1.24	0.042	0.86	0.67–1.11	0.245	1.14	1.02–1.27	0.022	0.92	0.72–1.17	0.492
Unemployed	0.59	0.40–0.87	0.009	0.89	0.81–0.97	0.007	0.91	0.66–1.25	0.547	1.08	0.97–1.20	0.150	1.25	1.09–1.43	0.002
Scheme															
Universal healthcare coverage	Ref			Ref			Ref			Ref			Ref		
Civil servant medical benefit	0.85	0.62–1.16	0.297	0.90	0.81–1.00	0.044	1.22	0.82–1.83	0.329	1.12	1.00–1.26	0.050	1.02	0.86–1.22	0.791
Social security	0.81	0.63–1.04	0.102	0.92	0.80–1.07	0.279	0.68	0.53–0.88	0.003	0.70	0.61–0.81	< 0.001	0.98	0.50–1.95	0.964
Others	0.86	0.47–1.60	0.640	0.82	0.63–1.06	0.133	0.83	0.46–1.50	0.533	0.81	0.62–1.05	0.113	0.98	0.53–1.82	0.958
Hypertension	4.18	3.50–4.99	< 0.001	5.58	5.22–5.97	< 0.001	4.80	3.98–5.80	< 0.001	5.26	4.90–5.65	< 0.001	6.42	0.53–7.32	< 0.001
Statin used	1.09	0.91–1.31	0.327	1.08	1.01–1.16	0.027	1.32	1.10–1.59	0.003	1.20	1.12–1.29	< 0.001	1.01	0.88–1.17	0.856
Fibrate used	1.63	1.22–2.19	0.001	1.37	1.22–1.54	< 0.001	1.41	1.10–1.82	0.008	1.41	1.24–1.60	< 0.001	1.79	1.30–2.46	< 0.001
Insulin used	0.97	0.79–1.19	0.788	1.04	0.97–1.12	0.297	0.93	0.77–1.13	0.481	0.95	0.88–1.03	0.237	0.96	0.81–1.14	0.635
Diabetic kidney disease	1.43	0.92–2.22	0.111	1.24	1.07–1.44	0.005	1.34	0.90–1.98	0.145	1.39	1.18–1.64	< 0.001	1.20	0.92–1.56	0.178

*Adjusted for TyG index, age, body mass index, region, scheme, hospital level, year, occupation, hypertension comorbidity, diabetic kidney disease, insulin used, fibrate used, and statin used.

**Adjusted for TyG index, sex, body mass index, region, scheme, hospital level, year, occupation, hypertension comorbidity, diabetic kidney disease, insulin used, fibrate used, and statin used.

*AOR* adjusted odds ratio, *CI* confidence interval, *TyG* triglyceride-glucose, *CVD* cardiovascular diseases.

## Discussion

The current study presents a nationwide serial cross-sectional study enrolling 63,815 Thai patients with T2D aged 30 to 74 years without history of CVD between 2014 and 2018. In our study with T2D populations, the mean TyG index values reached 9.30, which is relatively high in comparison with those of other related studies ranging from 8.50 to 9.20^17,23,24^. To our knowledge, our study is the first to examine the TyG index trends in T2D worldwide. A decreasing trend of the TyG index from 2014 to 2018 was demonstrated among both males and females. The observed reduction in the magnitude of the TyG index may still be minimal. Previous studies have demonstrated that changes in the TyG index across quartiles, or approximately 0.20–0.50 change in the TyG index, are associated with differences in CVD events^25,26^.

In the northeast region, a relatively high age- and sex-adjusted TyG index was observed, ranging from 9.40 to 9.43 in comparison with other regions with a range of 9.20–9.30. These results may be explained by the low physician-to-population ratio in the northeast region in comparison with other regions and their diet of glutinous rice^22^. Glutinous rice is also known to stimulate high triglyceride levels compared to nonglutinous rice^27^. The high TyG index was also associated with other cardiometabolic risk factors, including HT, metabolic syndrome, and other cardiovascular risks^4,14,20,28^. Therefore, improved glycemic and triglyceride levels should be focused on in northeast region populations.

Our study demonstrated an independent positive relationship between the TyG index and predicted 10-year CVD risk based on the FHS equation among patients with T2D. Similar to our results, Laura et al. indicated a nonlinear relationship between the TyG index and risk for CVD based on the FHS equation, but only among patients without HT or T2D at baseline^21^. The TyG index comprised FPG and TG levels, while the FHS equation included age, sex, SBP, history of diabetes, history of current smoking, and treatment for HT, TC, and HDL cholesterol. Prior studies demonstrated positive associations between the TyG index and several cardiometabolic risk factors such as dyslipidemia, hypertension, T2D, and smoking which were included in the FHS equation^14,24,29^. The mechanism explaining the TyG index and its association with CVD risk remains currently unclear. However, Tao et al. described three potential mechanisms between the TyG index and CVD. First, the TyG index is proven to be a surrogate of IR which in turn induces hyperglycemia causing inflammation and oxidative stress. Second, IR can escalate the production of glycosylated products and free radicals damaging endothelial function. Finally, IR also plays a vital role in hyperlipidemia. These issues may also result in the pathogenesis of atherosclerosis^4,30^.

A large cohort in China described sex-related differences in association with the TyG index and predicted CVD risk. A relatively higher association and predictive ability were found between the TyG index and predicted 10-year CVD risk among females compared to males^31^. Sex disparities in the relationship between other risk factors and CVD are now widely accepted. For instance, hyperuricemia increased the risk of coronary heart disease incidence and mortality among females, but not males^31,32^. Similarly, our study demonstrated a relatively stronger relationship between the TyG index and log-transformed predicted CVD risk among females compared to males. Meanwhile, the TyG index indicated similar predictive ability between male and female patients.

As previously known, older age was associated with an increased risk of CVD^6,33^. However, in our study, lower TyG index levels were observed in older age groups. A study on patients with hypertension in China also found similar results that the TyG index was inversely associated with age^34^. Sakboonyarat et al. reported that younger patients with T2D between 2011 and 2018 in Thailand tended to reveal poorer glycemic control in comparison with older patients probably due to motivation and adherence in T2D treatment including pharmacologic treatment, lifestyle modification, and diet restrictions^22^. Similarly, a large related study in Thailand demonstrated that the prevalence of hypertriglyceridemia continuously declines with higher age groups after reaching its peak level at 40–44 years^35^. However, age might not be the main contributor to uncontrolled hyperlipidemia among Thai patients with T2D^36^. These findings might affirm the need for an effective program to improve glycemic and triglyceride control among younger patients.

Our study is the first to formulate a cutoff point of the TyG index in predicting intermediate-to-high predicted 10-year CVD risk among patients with T2D. The cutoff point established was 9.20, with sensitivity of 55.7% and specificity of 46.8% with fair AUC (0.565). The cutoff point is relatively high, and its sensitivity and specificity were relatively low in comparison with similarly related studies. Susilane et al. established a cutoff point at 9.04 (AUC = 0.678, sensitivity of 62.5%, and specificity of 66.7%; *p* < 0.001) to predict intermediate-to-high CVD risk based on FHS among patients with cardiometabolic risk^20^. Luo et al. also determined a cutoff point for predicting cardiovascular events at > 9.00 among patients with a history of ST-elevated myocardial infarction. However, Wang et al. identified a value of 9.32 (AUC = 0.560, sensitivity of 46.0%, and specificity of 63.6%) for predicting major adverse cardiovascular events (MACE) among patients with T2D undergoing coronary angiography^24^. This may indicate that diabetic populations showed a higher cutoff point and lower sensitivity and specificity compared to non-diabetic populations. Consequently, the use of the TyG index to predict CVD risk among individuals with type 2 diabetes might be questionable.

Furthermore, after formulating the cutoff point at 9.20, we conveyed that a high TyG index of over 9.20 is an independent risk for having intermediate-to-high predicted 10-year CVD risk among patients with T2D (AOR = 2.50, 95% CI 2.35–2.66, *p* < 0.001). After stratifying by sex and age groups, both males and females and different age groups with a TyG index over 9.20 showed higher risk of having intermediate-to-high predicted 10-year CVD risk. The TyG index had previously been introduced as a potential marker in CVD, but data remain scarce regarding its ability to predict future CVD risk. Laura et al. included the TyG index in the Framingham variable to formulate a new model showing better prediction of CVD events in comparison with the usual FHS model. The study determined a higher AUC value (0.719) for the Framingham plus TyG index model in comparison with the usual FHS model (0.708, *p* = 0.014)^17,21^. Laura et al. also demonstrated that the TyG index was significantly associated with an increased risk of developing CVD but not among patients with T2D. However, they did not account for lipid-lowering or antidiabetics medication^21^. Our study concluded that a high TyG index is an independent risk factor of intermediate-to-high and high predicted 10-year CVD risk in both male and female patients with T2D in age groups of 30–74 years. Thus, the TyG index may provide additional predictive value to TG and FBG levels in identifying high-risk CVD patients with T2D.

The present study encountered several limitations. First, our study included only T2D patients receiving treatment in hospitals, excluding primary care units and university hospitals, which is considered about one-half of the overall patients with T2D in Thailand^22^. Second, our study employed a serial cross-sectional design; therefore, FPG and lipid profile levels were only measured at baseline. Consequently, the TyG index and predicted CVD risk might change over the course of diabetes or dyslipidemia treatment. A future prospective cohort study among the population could be beneficial in enhancing the predictive potential and adding value to the TyG index in predicting the CVD risk^37^. Moreover, due to the nature of an observational study, some variables, including SBP for 92 (0.1%) participants and BMI for 835 (1.3%) participants, were missing. However, due to having an extensive data set, we did not perform data imputation. Consequently, individuals with missing data were still included in the model.

Third, this study calculated the predicted CVD risk using the FHS data which was conducted in a US population. However, the score was validated in a retrospective cohort including multiethnic Asian populations, showing fairly accurate prediction for males and slightly overestimated prediction for females^38^. Nonetheless, a calibration study conducted on individuals with T2D in the UK suggested that CVD risk prediction scores might not precisely identify those who would experience a cardiovascular event within the 10-year follow-up period^39^. Hence, caution should be taken when interpreting the results as a CVD risk prediction tool for patients with T2D^40^. Fourth, the HOMA-IR value, which is another well-known insulin resistance surrogate, cannot be included due to the absence of serum insulin level. Finally, our study determined the cutoff point of the predicted 10-year CVD risk as its outcome. Hence, data on using the TyG index to predict CVD event outcomes should be further investigated among patients with T2D.

This study also exhibited several strengths. It is the first to formulate a cutoff point of the TyG index in predicting intermediate-to-high predicted 10-year CVD risk among patients with T2D, emphasizing the need to determine different cutoff points among patients with T2D. The trends of the TyG index in Thailand were also determined, especially among patients with T2D. Thus, our results provided beneficial awareness. Even though TyG index trends had decreased from 2014 to 2018, our TyG index is relatively high in comparison with related studies worldwide, empowering increasing attention to control FPG, TG, and cardiometabolic risk.

## Conclusion

Our study demonstrated decreased overall age- and sex-adjusted mean trends of the TyG index between 2014 and 2018. Nevertheless, these values are still high in comparison with related studies. Consequently, better glycemic and lipid control are needed. A high TyG index indicated an independent association with an increased predicted 10-year CVD risk among patients with T2D. However, the predictive ability of the TyG index in assessing the 10-year CVD risk remains questionable. Nevertheless, patients with T2D and a high TyG index should be encouraged to improve the control of their cardiovascular risk factors.

### Supplementary Information

Supplementary Information.

## Data Availability

Data cannot be shared publicly because the data set contains identifying information; additionally, the data belong to the Thailand DM/HT study of the MedResNet. Thus, ethics restrictions exist on the data set. Data are available from the Thai NHSO, Bangkok, Thailand (contact Sirikorn Khunsri via sirikorn@nhso.go.th) for researchers meeting the criteria to access confidential data.

## Abbreviations

NCDs: Noncommunicable diseases
CVD: Cardiovascular diseases
FHS: Framingham heart study
TyG index: Triglyceride-glucose index
T2D: Type 2 diabetes
BMI: Body mass index
SBP: Systolic blood pressure
HT: Hypertension
DKD: Diabetic kidney disease
CI: Confidence interval
SD: Standard deviation
IQR: Interquartile range
OR: Odds ratio
AOR: Adjusted odds ratio
