# Supplementary material for: Trends in baseline triglyceride-glucose index and association with predicted 10-year cardiovascular disease risk among type 2 diabetes patients in Thailand
Source: Sci Rep. 2023 Aug 10;13:12960. doi: 10.1038/s41598-023-40299-y (PMC10415402; doi:10.1038/s41598-023-40299-y)
Supplement: Supplementary file 1 — Supplementary Information. [file 41598_2023_40299_MOESM1_ESM.docx]

**Supplementary file**

**Trends in Baseline Triglyceride-Glucose Index and Association with Predicted 10-Year Cardiovascular Disease Risk among Type 2 Diabetes Patients in Thailand**

Sethapong Lertsakulbunlue^1^, Mathirut Mungthin^2^, Ram Rangsin^3^, Anupong Kantiwong^1^ *Boonsub Sakboonyarat^3^

^1^Department of Pharmacology, Phramongkutklao College of Medicine, Bangkok 10400, Thailand

^2^Department of Parasitology, Phramongkutklao College of Medicine, Bangkok 10400, Thailand

^3^Department of Military and Community Medicine, Phramongkutklao College of Medicine, Bangkok 10400, Thailand

| **Title** | **Page number** |
| --- | --- |
| **Supplementary Figure 1.** ROC curve of the TyG index to predict intermediate-to-high 10-year predicted CVD risk among female participants. | 2 |
| **Supplementary Figure 2.** ROC curve of the TyG index to predict intermediate-to-high 10-year predicted CVD risk among male participants. | 3 |
| **Supplementary table 1.** Univariable logistic regression analysis for factor associated with intermediate to high predicted CVD risk among T2D patients without clinical CVD**.** | 4 |
| **Supplementary table 2.** Logistic regression analysis of high CVD risk and Triglyceride-glucose index**.** | 5 |

**Supplementary Figure 1.** ROC curve of the TyG index to predict intermediate-to-high 10-year predicted CVD risk among female participants.

**
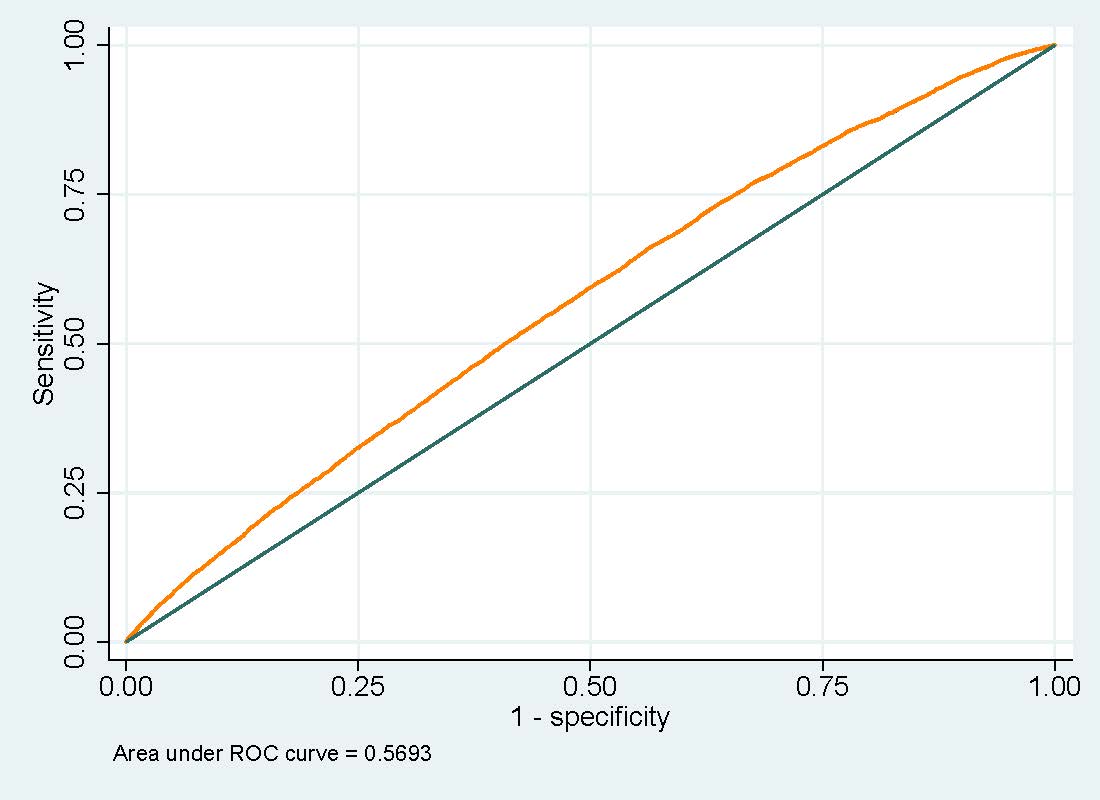
**

**Supplementary Figure 2.** ROC curve of the TyG index to predict intermediate-to-high 10-year predicted CVD risk among male participants.


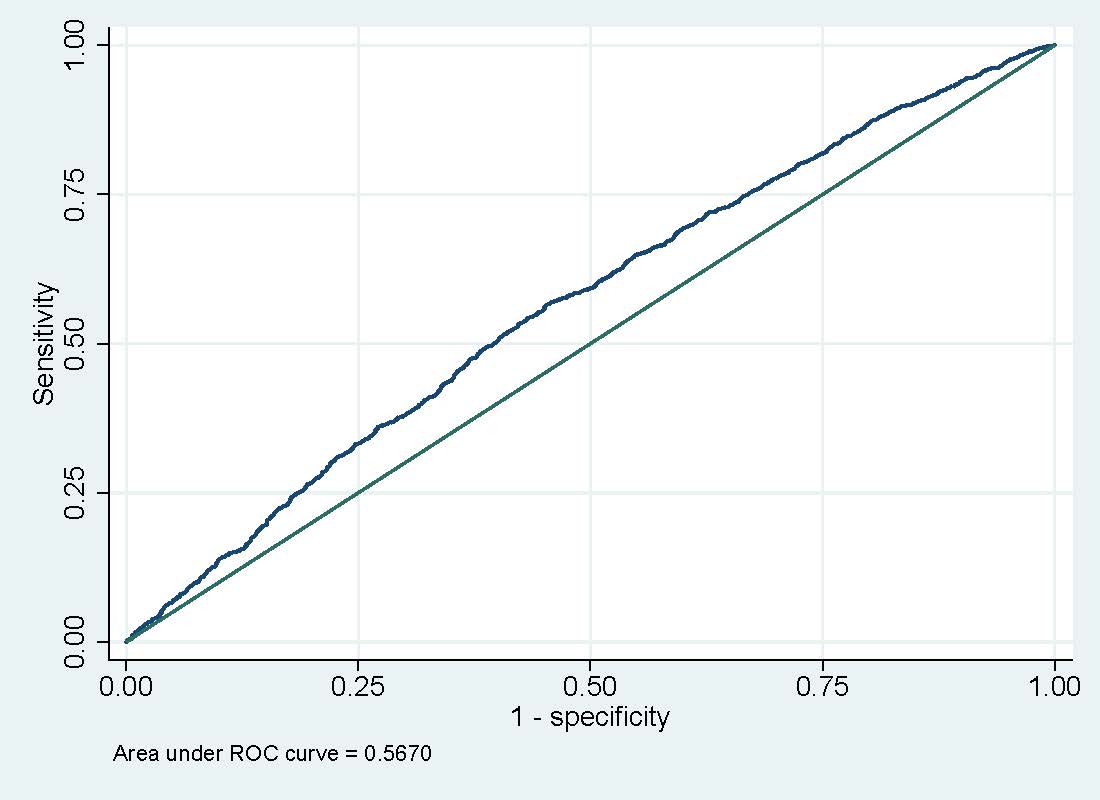


| Supplementary table 1. Univariable logistic regression analysis for factor associated with intermediate to high predicted CVD risk among T2D patients without clinical CVD | | | | | |
| --- | --- | --- | --- | --- | --- |
| **Factors** | **CVD risk** | | **Crude OR** | **95% CI** | ***p*-value** |
|  | **10% n(%)** | **≥10% n(%)** |  |  |  |
| **Sex** |  |  |  |  |  |
| Male | 1181 (11.0) | 18054 (35.4) | ref |  |  |
| Female | 9528 (89.0) | 32902 (64.6) | 0.23 | 0.21-0.24 | <0.001 |
| **Age mean±S.D.** | 49±8.3 | 61±7.7 | 1.19 | 1.19-1.20 | <0.001 |
| **Year** |  |  |  |  |  |
| 2014 | 3530 (33.0) | 14522 (28.5) | ref |  |  |
| 2015 | 3188 (29.8) | 15588 (30.6) | 1.19 | 1.13-1.25 | <0.001 |
| 2018 | 3991 (37.3) | 20846 (40.9) | 1.27 | 1.21-1.33 | <0.001 |
| **Geographic region** |  |  |  |  |  |
| Northeast | 1719 (16.1) | 8114 (15.9) | ref |  |  |
| North | 3222 (30.1) | 17125 (33.6) | 1.13 | 1.06-1.20 | <0.001 |
| Central | 3769 (35.2) | 15436 (30.3) | 0.87 | 0.81-0.92 | <0.001 |
| South | 1719 (16.1) | 8408 (16.5) | 1.04 | 0.96-1.12 | 0.342 |
| Bangkok | 280 (2.6) | 1873 (3.7) | 1.42 | 1.24-1.62 | <0.001 |
| **Hospital level** |  |  |  |  |  |
| Regional hospital | 837 (7.8) | 3953 (7.8) | ref |  |  |
| General hospital | 1546 (14.4) | 7881 (15.5) | 1.08 | 0.98-1.18 | 0.105 |
| Community & Health Promoting hospital | 8326 (77.7) | 39122 (76.8) | 0.99 | 0.92-1.08 | 0.898 |
| **Occupation** |  |  |  |  |  |
| Outdoor occupation | 7650 (73.3) | 29308 (59.3) | ref |  |  |
| Indoor occupation | 1338 (12.8) | 6554 (13.3) | 1.28 | 1.20-1.36 | <0.001 |
| Unemployed | 1445 (13.9) | 13578 (27.5) | 2.45 | 2.31-2.60 | <0.001 |
| **Scheme** |  |  |  |  |  |
| Univeral healthcare coverage | 8566 (80.0) | 39713 (78.0) | ref |  |  |
| Civil servant medical benefit | 987 (9.2) | 8639 (17.0) | 1.89 | 1.76-2.03 | <0.001 |
| Social security | 965 (9.0) | 1820 (3.6) | 0.41 | 0.38-0.44 | <0.001 |
| Others | 185 (1.7) | 758 (1.5) | 0.88 | 0.75-1.04 | 0.136 |
| **BMI (kg/m2)** |  |  |  |  |  |
| <18.5 | 396 (3.7) | 1406 (2.8) | ref |  |  |
| 18.5-22.9 | 2570 (24.2) | 11648 (23.1) | 1.28 | 1.13-1.44 | <0.001 |
| 23-24.9 | 1959 (18.5) | 10322 (20.5) | 1.48 | 1.31-1.68 | <0.001 |
| 25-29.9 | 3713 (35.0) | 18765 (37.2) | 1.42 | 1.27-1.60 | <0.001 |
| ≥30 | 1970 (18.6) | 8255 (16.4) | 1.18 | 1.04-1.33 | 0.008 |
| **Hypertension** |  |  |  |  |  |
| No | 5891 (55.0) | 9341 (18.3) | ref |  |  |
| Yes | 4818 (45.0) | 41615 (81.7) | 5.45 | 5.21-5.69 | <0.001 |
| **Diabetic kidney disease** |  |  |  |  |  |
| No | 10300 (96.2) | 47162 (92.6) | ref |  |  |
| Yes | 409 (3.8) | 3794 (7.4) | 2.03 | 1.83-2.24 | <0.001 |
| **Insulin use** |  |  |  |  |  |
| No | 8191 (76.5) | 39789 (78.1) | ref |  |  |
| Yes | 2518 (23.5) | 11167 (21.9) | 0.91 | 0.87-0.96 | <0.001 |
| **Fribate use** |  |  |  |  |  |
| No | 9789 (91.4) | 44919 (88.2) | ref |  |  |
| Yes | 920 (8.6) | 6037 (11.8) | 1.43 | 1.33-1.54 | <0.001 |
| **Statin use** |  |  |  |  |  |
| No | 4467 (41.7) | 17882 (35.1) | ref |  |  |
| Yes | 6242 (58.3) | 33074 (64.9) | 1.32 | 1.27-1.38 | <0.001 |
| SD: standard deviation; BMI: body mass index; CVD: cardiovascular disease. | | | | | |

| Supplementary table 2. Logistic regression analysis of high CVD risk and Triglyceride-glucose index | | | | | | |
| --- | --- | --- | --- | --- | --- | --- |
| **High predicted 10-year CVD risk** | | | | | | |
| **Variables** | **Univariate** | | | **Multivariate*** | | |
|  | **OR** | **95% CI** | ***p*-value** | **AOR** | **95% CI** | ***p*-value** |
| **TyG index** | 1.46 | 1.43-1.50 | <0.001 | 2.67 | 2.56-2.78 | <0.001 |
| **TyG index** |  |  |  |  |  |  |
| TyG index ≤9.2 | ref |  |  | ref |  |  |
| TyG index >9.2 | 1.45 | 1.40-1.50 | <0.001 | 2.51 | 2.39-2.63 | <0.001 |
| **TyG index (Quartiles)** |  |  |  |  |  |  |
| Quartile 1 (<8.86) | ref |  |  | ref |  |  |
| Quartile 2 (8.86-9.26) | 1.24 | 1.19-1.30 | <0.001 | 1.70 | 1.60-1.81 | <0.001 |
| Quartile 3 (9.26-9.69) | 1.45 | 1.39-1.52 | <0.001 | 2.56 | 2.40-2.73 | <0.001 |
| Quartile 4 (>9.69) | 1.80 | 1.72-1.88 | <0.001 | 4.59 | 4.28-4.91 | <0.001 |
| *Adjusted for age, sex, body mass index, region, scheme, hospital level, year, occupation, hypertension comorbidity, diabetic kidney disease, insulin used, fibrate used, and statin used. OR: odds ratio; AOR: adjusted odds ratio; CVD: cardiovascular disease; TyG: triglyceride-glucose; CI: confidence interval. | | | | | | |
